# Medial patellofemoral ligament reconstruction with a synthetic polyester suture tape graft and knotless anchors: Five-year clinical and functional outcomes

**DOI:** 10.1051/sicotj/2026010

**Published:** 2026-04-15

**Authors:** Wessam Fakhery Ebied, Mohamed Ashry, Mohamed Amr Hemida, Ahmed H. Khater, Mohamed H. Sobhy, Yahia Haroun

**Affiliations:** 1 Orthopedic Department, Faculty of Medicine, Ain Shams University 38 Abbassia 11566 Cairo Egypt; 2 Saudi German Hospital Dammam Airport Road, Dammam, Eastern province. P.O Box 2550 Dammam 21461 KSA

**Keywords:** Patellar Dislocation, suture tape, Knotless anchor, Medial patellofemoral ligament

## Abstract

*Introduction*: Medial patellofemoral ligament (MPFL) reconstruction is a well-established treatment for recurrent lateral patellar dislocations, yielding satisfactory clinical outcomes. Although synthetic materials are not widely used due to limited long-term data, they offer the potential to eliminate donor-site complications and may provide promising results. This study evaluated the five-year clinical and functional outcomes of MPFL reconstruction using suture tape, hypothesising that it is a safe alternative to traditional grafts. *Methods*: Thirty patients aged 20 – 45 years with recurrent lateral patellar dislocations were treated between 2017 and 2020. Exclusion criteria included patellofemoral joint pathology, high-grade trochlear dysplasia, patella alta, neuromuscular disorders, or significant lower limb malalignment requiring correction. All patients underwent MPFL reconstruction using suture tape, placed in the superomedial half of the patella and fixed to the femoral footprint using a knotless anchor. The vastus medialis obliquus insertion was advanced laterally and distally. Preoperative assessments included clinical examinations, knee radiographs, alignment views, TT-TG measurements via CT scans, and MRIs. Patients were evaluated using the Kujala scale, International Knee Documentation Committee (IKDC) score, Crosby and Insall grading system, and Lysholm score. *Results*: At the 5-year follow-up, all patients had resumed their daily activities without recurrence of dislocation. The mean Kujala score improved from 65.23 to 93.60 (*P* < 0.001), with significant increases also observed in IKDC and Lysholm scores (*P* < 0.001). According to the Crosby/Insall grading system, 24 patients were rated as “excellent”, and six patients were rated as ‘good’. The mean knee extension was −5°, and flexion was 140° at the final follow-up. *Conclusion*: MPFL reconstruction using suture tape with knotless anchors, combined with careful patient selection, appears to be a safe and effective option, demonstrating satisfactory five-year clinical outcomes and no recurrence of instability. However, this study was limited by its relatively small sample size and retrospective design.

## Introduction

The medial patellofemoral ligament (MPFL) is the primary restraint against lateral patellar translation between 0° and 30° of flexion [1]. It is invariably injured in patellofemoral instability [2]. Therefore, MPFL reconstruction is a logical procedure for recurrent lateral patellar dislocations, either alone or in conjunction with other procedures, such as tibial tuberosity osteotomy [3, 4].

Various MPFL reconstruction techniques have been reported, which differ in patellar and femoral fixation methods and the type of graft used [5–8]. Graft options include autografts (the classic choice), allografts, and synthetic grafts [5–8]. Although not yet a popular choice, artificial grafts avoid sacrificing autologous tissue, eliminate donor-site morbidity, and have demonstrated promising clinical results [3, 6, 9, 10].

Autograft hamstring tendons remain the most frequently used grafts for MPFL reconstruction [5], but synthetic options have re-emerged due to their favourable biomechanical properties and the absence of donor-site morbidity. Modern suture tapes exhibit high initial stiffness and low elongation, and biomechanical studies have shown superior load-to-failure characteristics compared to autograft constructs [11, 12]. Clinical studies and systematic reviews have also reported outcomes comparable to those of autograft techniques [13–15]. Despite these advantages, suture tapes remain less widely adopted, partly due to historical concerns about early synthetic ligament failures and scarcity of long-term data [16, 17].

This study aimed to evaluate the five-year clinical and functional outcomes of MPFL reconstruction using synthetic polyester suture tape (FiberTape; Arthrex^®^, Naples, FL, USA) and knotless anchors (SwiveLock; Arthrex^®^). We hypothesised that suture tape is a safe and reliable alternative for MPFL reconstruction.

## Materials and methods

This retrospective study included 30 patients with recurrent patellar dislocations who were treated at Ain Shams University Hospitals in Cairo, Egypt, between January 2017 and January 2020. The inclusion criterion was patient age 20–45 years.

Suture tape was routinely used in this study to assess its performance as a primary graft option in appropriately selected patients. Suitability was determined based on the following criteria:

1. Exclusion criteria: patellofemoral joint pathology (arthritis, fixed dislocations, congenital dislocations, habitual dislocations), high-grade trochlear dysplasia (Dejour 3 or 4), patella alta (Caton–Deschamps Index (CDI) > 1.3), neuromuscular disorders, or significant skeletal lower limb malalignment such as valgus or recurvatum >10°, increased femoral anteversion > 45°, increased external tibial rotation, or tibial tuberosity to trochlear groove distance (TT-TG) > 20 mm that would require additional surgical correction.
2. Patient preference and informed consent: Patients were informed of the advantages (no donor-site morbidity and shorter operative time) and limitations (lack of long-term data) of suture-tape grafts. All participants explicitly consented to the use of suture tape.

Ethical considerations were addressed by obtaining approval from the Research Ethics Committee.

Patients’ preoperative demographics were recorded. They were evaluated using history and clinical examination, in addition to standard knee radiographs, long-film alignment views, CT measuring the TT-TG distance, and MRI.

Patients were evaluated preoperatively using the Kujala score, International Knee Documentation Committee (IKDC) Subjective Knee Evaluation, Crosby and Insall grading system, Tegner Lysholm Knee Score, and knee range of motion.

## Operative technique

**Figure 1:**
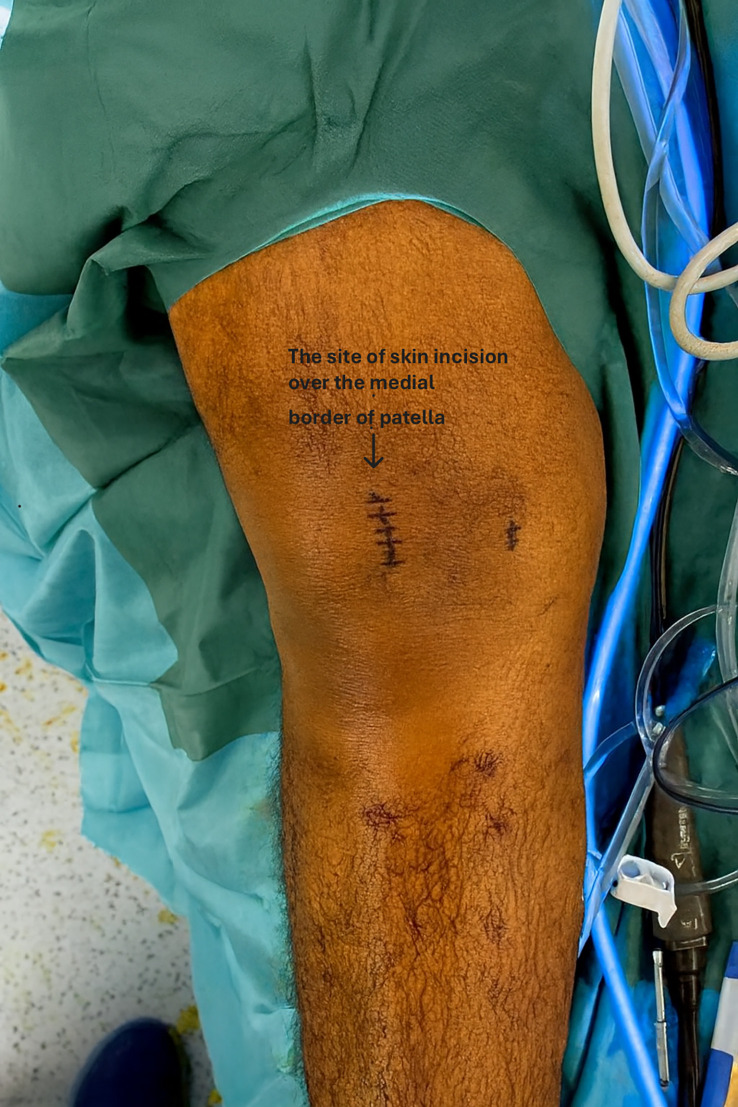
Site of the skin incision over the medial border of patella.

All patients underwent knee examination under anaesthesia and were operated on in the supine position with a tourniquet. Standard knee arthroscopy was performed. A skin incision was made along the medial patellar border (Figure 1). Dissection was performed from the subcutaneous layer to layer 1.

The vastus medialis obliquus (VMO) muscle was identified, and a medial longitudinal parapatellar incision was made to expose layer 2. The incision was extended proximally along the VMO insertion, which was later used for imbrication during closure.

**Figure 2:**
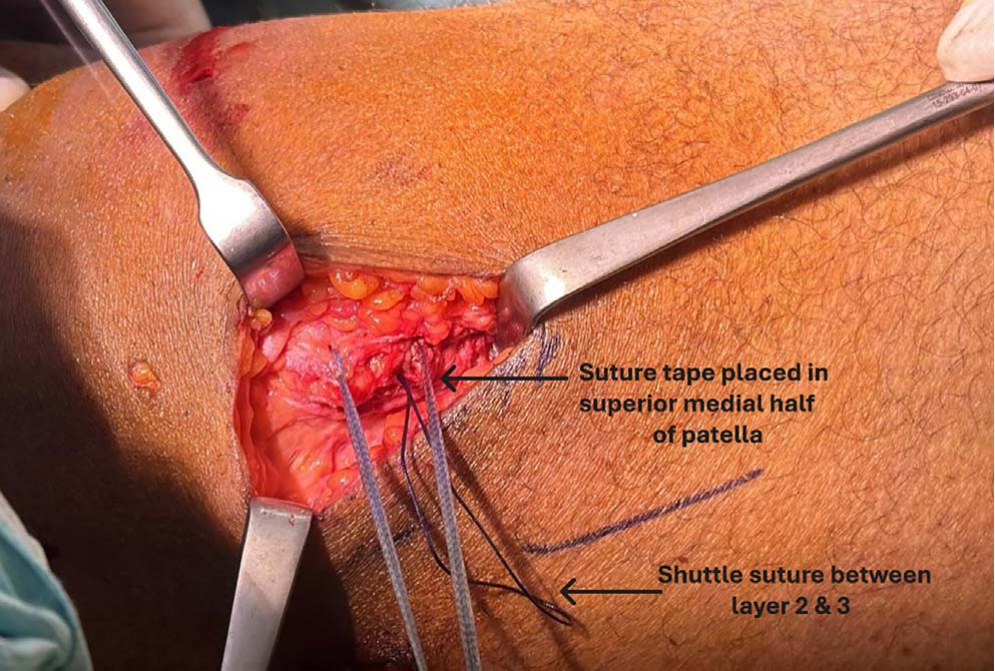
Suture tape placed into the prepatellar soft tissue and periosteum.

**Figure 3:**
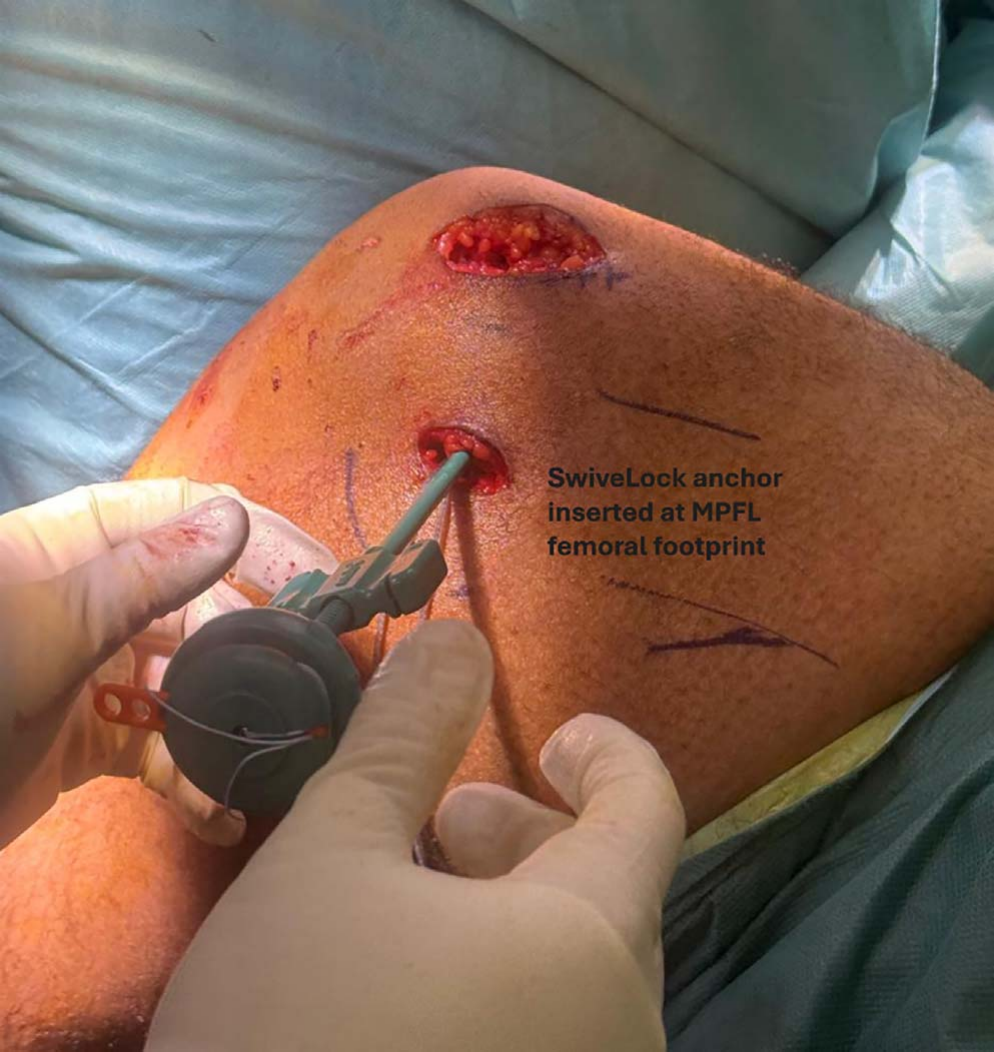
Fixation of suture tape with a knotless anchor at the femoral footprint of the MPFL.

The medial patellar soft-tissue sleeve was dissected from the patella, and a suture tape (Arthrex^®^, Naples, FL) was placed in the superior half, incorporating the prepatellar periosteum for secure fixation (Figure 2). This provided hardware-free patellar-side fixation at a lower cost. A virtual extra-synovial space was created via blunt dissection between layers 2 and 3 towards the femoral origin of the MPFL. A small incision over the medial femoral condyle was made to access the MPFL footprint, known as Schöttle’s point. The position was checked using an image intensifier [18]. The two free ends of the suture tape were shuttled from the medial border of the patella to the femoral footprint of the MPFL in the second layer, positioned superficial to the synovium. Under fluoroscopic guidance, a 1.8‑mm K‑wire was inserted into Schöttle’s point. The suture tape was looped around the wire, and the knee was taken through a full range of motion to confirm that the femoral attachment corresponded to the isometric point. A SwiveLock knotless anchor (Arthrex^®^, Naples, FL) was then used to secure the suture tape at the femoral insertion, with the patella centred within the trochlear groove at 30° flexion and without excessive tension (Figure 3). Correct positioning of the femoral footprint was essential to avoid over‑tensioning the reconstructed MPFL. Tension assessment included confirming two quadrants of lateral patellar translation, cycling the knee from 0° to 90° to assess patellar tracking, ensuring that the patella remained engaged within the trochlea without excessive lateral translation in extension, and securing the anchor at 30° flexion. The optimal flexion angle during graft fixation is debatable, with a general recommendation of 30° [19]. It is important to avoid excessive tension, as it can lead to pain, stiffness, and quadriceps inhibition [20]. The VMO insertion was then advanced 5–10 mm laterally and distally on the prepatellar soft tissue using no. 2 non-absorbable sutures (ETHIBOND; Ethicon^®^, Somerville, NJ), placed in a pants-over-vest fashion. This was performed routinely in all patients to enhance dynamic medial stabilisation and optimise patellar tracking, thereby complementing the static restraint provided by the reconstructed ligament. Subcutaneous and skin closures were then performed, and the knee was placed in a hinged brace. Antibiotic prophylaxis was continued for the first 24 h.

## Postoperative rehabilitation

Immediately after surgery, patients were encouraged to use ice packs and perform ankle pumps. For the first four weeks, the extremity was protected by a hinged knee brace. Patients were allowed to weight-bear as comfortably as possible with crutches and to perform passive and assisted active range-of-motion exercises as tolerated. From 5 to 10 weeks, patients discontinued use of the brace and began active range-of-motion and assisted-strengthening exercises. From 11 to 16 weeks, patients gradually regained a full range of motion and began muscle strengthening.

## Follow-up and outcomes

Patients were followed up at 2 weeks, 6 weeks, 3 months, and 6 months postoperatively, and then annually for 5 years. At each follow-up, patients underwent clinical assessment and were re-scored using the same functional scores as in the preoperative assessment (Kujala score, IKDC Subjective Knee Evaluation, Crosby and Insall grading system, Tegner Lysholm Knee Score, and knee range of motion). Any complications were recorded.

## Statistical Analysis

Data were analysed using an IBM-compatible PC with Statistical Package for the Social Sciences version 26.0.0, Microsoft Office Excel 2010, and GraphPad Prism 6. Descriptive statistics were calculated for the study group and are presented as mean, median, standard deviation, minimum, maximum, range, and percentages. Normality of quantitative data was assessed using the Shapiro-Wilk test.

For parametric data, comparisons between paired groups were performed using the paired t‑test. The level of statistical significance was set at *P* < 0.05, with results interpreted as follows:

*P* > 0.05 = non-significant

*P* < 0.05 = significant

*P* < 0.001 = highly significant

## Results

Thirty patients (17 males and 13 females, with a mean age of 22.8 years) met the inclusion and exclusion criteria; their demographics are presented in Table 1. All patients were followed up for five years.

**Table 1:** Demographic data of the studied patients.

Age at surgery (years)	Mean ± SD	22.83 ±3.98
	Range	17 – 33
Sex (*n*, %)	Female	13 (43.3%)
	Male	17 (56.7%)

The preoperative mean Kujala score was 65.23 ± 5.75, which significantly increased to 93.60 ± 3.64 (*P* < 0.001). All patients showed significant functional improvements in the IKDC and Lysholm scores (*P* < 0.001).

According to the Crosby/Insall grading system, 24 patients were graded as excellent, and the remaining patients were graded as good. The mean knee extension was -5 ° (−10° to 0°), and the mean knee flexion was 140° (130°−150°). At the final follow-up, all patients had returned to their previous daily living activities with no recurrence of dislocation (Table 2).

**Table 2:** Comparison of function scores between preoperative measurements and the latest follow-up values.

Parameter	Preoperative	Latest follow-up	*P-*value
	Mean ± SD	Mean ± SD	
	Range	Range	
Kujala score	65.23 ± 5.75	93.60 ± 3.64	< 0.001
	57 – 76	86 – 98	
IKDC	62.23 ± 8.87	90.70 ± 4.86	< 0.001
	44 − 80	78 − 98	
Lysholm score	53.37 ± 7.42	86.03 ± 4.33	< 0.001
	44 − 72	76 − 89	

Regarding complications, six patients experienced paraesthesia in the infrapatellar region, which resolved within one year. Four patients reported medial-sided pain at the femoral fixation site, which also resolved within one year. One patient had a superficial wound infection that resolved with antibiotics. Five patients reported audible clicking with knee flexion, which resolved within one year (Table 3).

**Table 3:** Postoperative complications, reoperations, and adverse events.

Event type	Number of cases	Description/outcome	Time to resolution
Infrapatellar paresthesia	6	Transient sensory disturbance	Resolved ≤ 1 year
Medial femoral fixation site pain	4	Localized pain at the anchor site	Resolved ≤ 1 year
Superficial wound infection	1	Managed with oral antibiotics, no sequelae	Resolved ≤ 1 year
Audible clicking with knee flexion	5	Mechanical clicking during flexion	Resolved ≤ 1 year
Reoperations	0	No revision or secondary procedures required	–
Major adverse events	0	No graft failure, instability, or major events	–

## Discussion

The primary finding of this study was that MPFL reconstruction using suture tape with knotless femoral fixation yielded effective five-year clinical outcomes. Specifically, there was no recurrent instability, and significant improvements were observed in mean Kujala, Lysholm, Crosby/Insall, and IKDC scores across all patients. These results are comparable to, and in some respects exceed, the outcomes reported for autograft-based MPFL reconstructions.

Several large autograft series have reported recurrent instability rates of 4.8%–7.1% after isolated MPFL reconstruction [21], even with anatomic tunnel placement and modern fixation techniques. However, no patient in this study experienced recurrent dislocation or persistent apprehension at five years. Furthermore, functional improvements were consistent with or slightly higher than those reported in autograft studies, in which postoperative Kujala scores typically ranged from the high 80s to the low 90s [5, 7]. These results suggest that suture tape provides comparable stability and functional recovery while avoiding donor‑site morbidity and graft harvest-related pain or weakness.

The biomechanical characteristics of modern synthetic constructs may partially explain these outcomes. Suture tape demonstrates high initial stiffness and resistance to elongation. Biomechanical studies have shown superior load‑to‑failure properties compared with semitendinosus autograft constructs [12]. Similarly, Zimmermann et al. reported that soft‑tissue fixation using non-absorbable suture tape provides primary stability exceeding that of the native MPFL [11]. Contemporary analyses of knotless anchor systems have also shown improved resistance to slippage and more consistent fixation strength than traditional interference screw techniques [22]. These biomechanical advantages are consistent with our clinical findings, as no patient demonstrated progressive lateralisation or recurrent instability during follow‑up.

Our findings align with recent clinical studies and systematic reviews demonstrating that synthetic grafts achieve outcomes comparable to those of autograft or allograft MPFL reconstruction. Migliorini et al. reported satisfactory outcomes and complication rates similar to those of autograft techniques [14], while McNeilan et al. found no significant differences in recurrent instability or patient-reported outcomes across graft types [15]. Akhtar et al. further indicated that suture tape constructs offer biomechanical advantages and comparable or superior clinical outcomes [23]. Additionally, a prospective comparative study by Lee et al. demonstrated no significant differences between synthetic grafts and gracilis autografts [13]. The absence of recurrent instability in our cohort highlights the potential advantage of resisting graft elongation over time, although this requires confirmation in larger comparative studies.

Recent multicentre evidence supports the effectiveness of isolated MPFL reconstruction across various anatomical variations [24]. Feller et al. reported acceptable outcomes even with higher thresholds for patella alta, regardless of TT-TG distance or trochlear dysplasia, when the MPFL was reconstructed anatomically and tensioned correctly. Their international cohort revealed that isolated MPFL reconstruction remains reliable despite anatomical risk factors that often indicate combined procedures. These findings underscore the crucialness of precise surgical technique and graft isometry, aligning with our results, in which careful patient selection and femoral fixation led to excellent five-year stability. Although our study excluded high-grade dysplasia or significant malalignment, Feller et al. suggested that modern MPFL reconstruction methods, including synthetic constructs, may have broader applicability [24].

Despite these advantages, synthetic grafts are not widely adopted, likely due to several factors. First, early synthetic ligament systems were associated with synovitis, graft rupture, and high revision rates, creating a lasting negative perception among surgeons [16, 17]. Second, cost considerations may influence implant selection in some healthcare systems. Third, although modern synthetic materials differ substantially from earlier designs, long-term clinical data beyond 10 years remain limited; thus, many surgeons continue to prefer autograft techniques because of their familiarity and established long-term outcomes. These factors collectively contribute to the slower uptake of synthetic options, despite growing evidence supporting their safety and efficacy [16, 17].

Although this series had a low complication rate and all issues were resolved without long-term morbidity, the use of synthetic grafts requires a more detailed consideration of potential risks. Modern polyester tapes have demonstrated improved biocompatibility compared with earlier generations of synthetic ligaments. Histological studies have shown fibroblast ingrowth and collagen deposition rather than the foreign‑body reactions historically associated with older materials [25, 26]. This could explain the absence of major graft-related complications in our study. Nevertheless, synthetic constructs carry theoretical risks. Foreign‑body inflammatory responses, graft elongation, and late mechanical fatigue have been reported in earlier synthetic ligament systems [16, 17]. Although these complications were not observed in our series, they are relevant concerns when interpreting mid-term outcomes. Stiffness and anterior knee pain have also been noted after MPFL reconstruction, regardless of graft type, and may be exacerbated by over-tensioning or non-anatomic femoral placement [7, 20]. Chronic medial-sided pain at the femoral fixation site has been reported in synthetic graft studies [27], consistent with the transient discomfort experienced by four patients in our study. Crucially, no cases of synovitis, graft failure, or persistent instability were observed, suggesting that modern suture tape constructs can mitigate many biological and mechanical complications historically associated with synthetic ligaments. However, longer-term follow-up is necessary to ascertain whether these promising mid-term outcomes persist beyond five years.

This study had some limitations, including its retrospective design, relatively small sample size, and the lack of a control group using autograft or allograft tissue. Nonetheless, the consistent improvement across all functional metrics, absence of recurrent instability, and low complication rates support the viability of suture tape as a primary graft option in appropriately selected patients.

Our operative technique is minimally invasive, reproducible, and anatomical. Additionally, we did not use fixation devices to secure the tape to the patella. We used only one anchor for femoral fixation, which reduced the overall cost of the implants. We recommend larger, well-designed, randomised controlled trials to compare the current suture technique with traditional fixation methods using autografts or allografts.

## Conclusion

MPFL reconstruction using suture tape with knotless anchors appears safe and effective at the five-year follow-up, with no recurrent instability. However, these findings are limited by the small sample size, retrospective design, and lack of a control group. Although our study excluded patients with multiple ligament injuries or prior hamstring harvest, this technique may offer theoretical advantages, such as avoidance of donor-site morbidity, preservation of autograft options, and applicability to more complex patient groups.

## Data Availability

The datasets used and/or analysed during the current study are available from the corresponding author upon reasonable request.
